# Role of ultrasound and colored Doppler examination in the diagnosis and the classification of the superficial soft tissue vascular anomalies

**DOI:** 10.1186/s43055-022-00753-9

**Published:** 2022-04-13

**Authors:** Asmaa Hussein Ibrahim Habib, Khalid Helmy El-Kaffas, Ahmed Sayed Mustafa, Shady Nabil Mashour

**Affiliations:** 1 Department of Diagnostic and Interventional Radiology, Faculty of Medicine, Cairo University, 1 Kasr El-ainy Street From El-kalig, Cairo, Egypt; 2 Ultrasonography and Doppler Unit, Department of Diagnostic and Interventional Radiology, Faculty of Medicine, Cairo University, 1 Kasr El-Ainy Street From El-kalig, Cairo, Egypt; 3 Department of Vascular Surgery, Faculty of Medicine, Cairo University, 1 Kasr El-Ainy Street From El-kalig, Cairo, Egypt

**Keywords:** Vascular anomalies, Ultrasound, Doppler, Infantile hemangiomas, Venous, Lymphatic, Arterial malformations

## Abstract

**Background:**

Vascular anomalies are congenital lesions of abnormal vascular development, and a primary distinction have to be made between a vascular tumor and a vascular malformation, hemangiomas are considered the commonest vascular tumor, correct diagnosis is imperative for appropriate treatment. In this report, we tried to verify the role of ultrasonography and Doppler examination in the initial diagnosis, the classification of vascular anomalies and in the post-treatment follow-up.

**Results (main findings):**

This report included cases of vascular anomalies who attended the interventional radiology department as well as the vascular anomaly clinic in Abo El-Rish hospitals during the period 2019 through 2021. Data of all patients attending the clinic were prospectively examined. Files of 60 cases with vascular anomalies were available for review. The diagnosis of vascular anomalies was done according to their history and characteristic findings at clinical examination as well as U/S and color Doppler examinations, MRI and angiographic studies were done as needed. A significant female predominance was noticed. A significant predominance in the head and neck region was noticed (60%). Treatment was individualized according to each case; propranolol was chosen as the first line of treatment in IH. Intra-lesional steroids injections were done in hemangiomas, and intra-lesional bleomycin was done in venous and lymphatic malformations, endovascular embolization was done in high flow vascular malformations.

**Conclusion:**

Ultrasound and color Doppler examination were effective and accurate methods in the diagnosis, the classification of superficial soft tissue vascular anomalies, the detection of early complications and in the follow-up after different treatment methods applied, it was also beneficial in the exclusion of non-vascular lesions.

## Background section

Vascular anomalies most commonly present in childhood by one of three manifestations: a cutaneous lesion with or without a characteristic appearance, a deeper palpable soft-tissue mass without diagnostic cutaneous features or a secondary clinical expression due to a recognized malformative syndrome [1].

Imaging may be employed if the deep extent of the lesion is unclear (such as a peri-orbital cutaneous abnormality that suggests the presence of an intra-orbital component that could ultimately impair vision) or its distribution implies a significant associated anomaly (such as a lesion overlying the lumbar spine that suggests an underlying tethering of the spinal cord) [2].

**Fig. 1:**
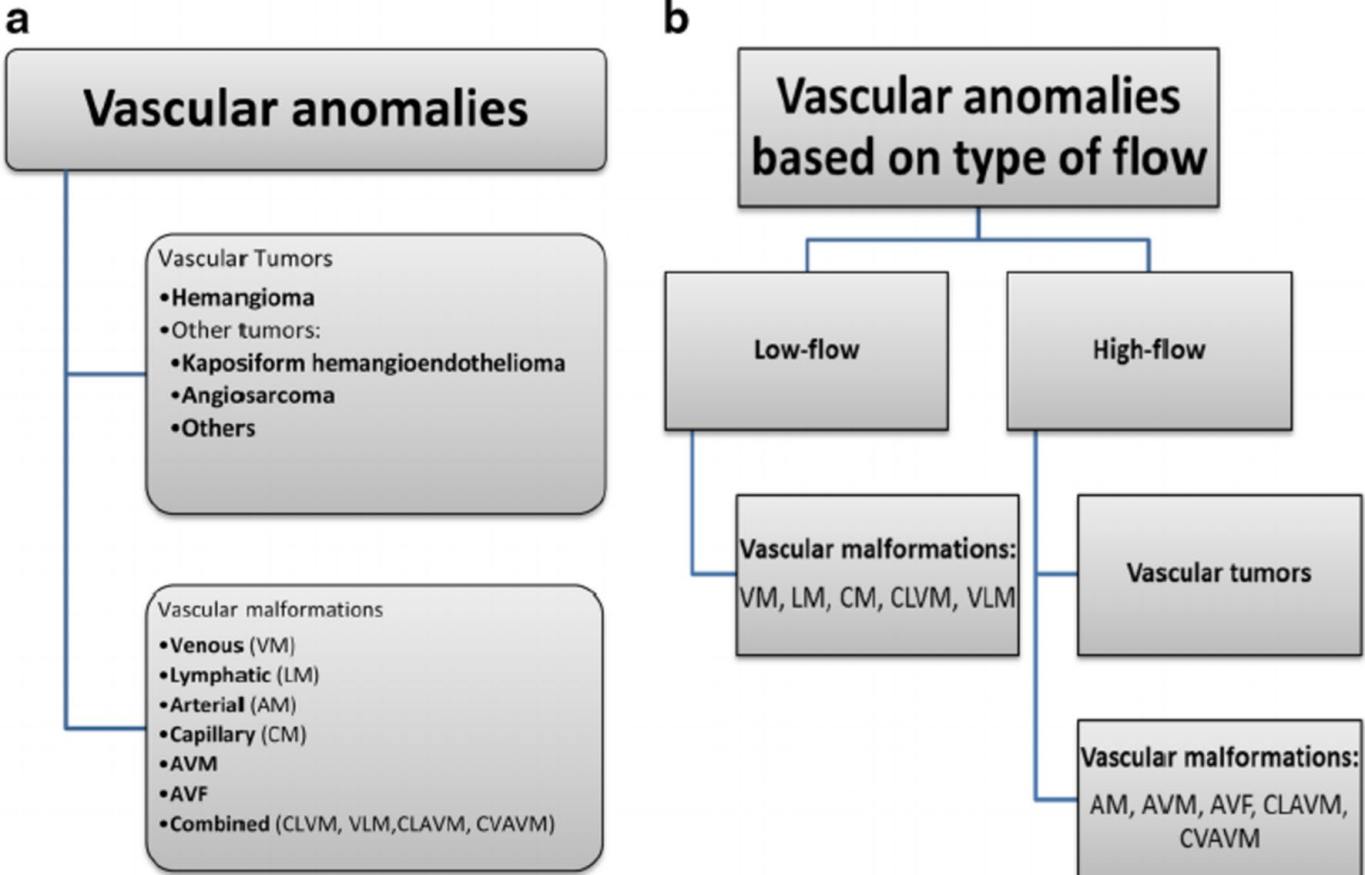
Classification of vascular anomalies. (Adapted from the International Society for the Study of Vascular Anomalies. “International Society for the Study of Vascular Anomalies (2014) ISSVA classification updates [4]”

**Fig. 2:**
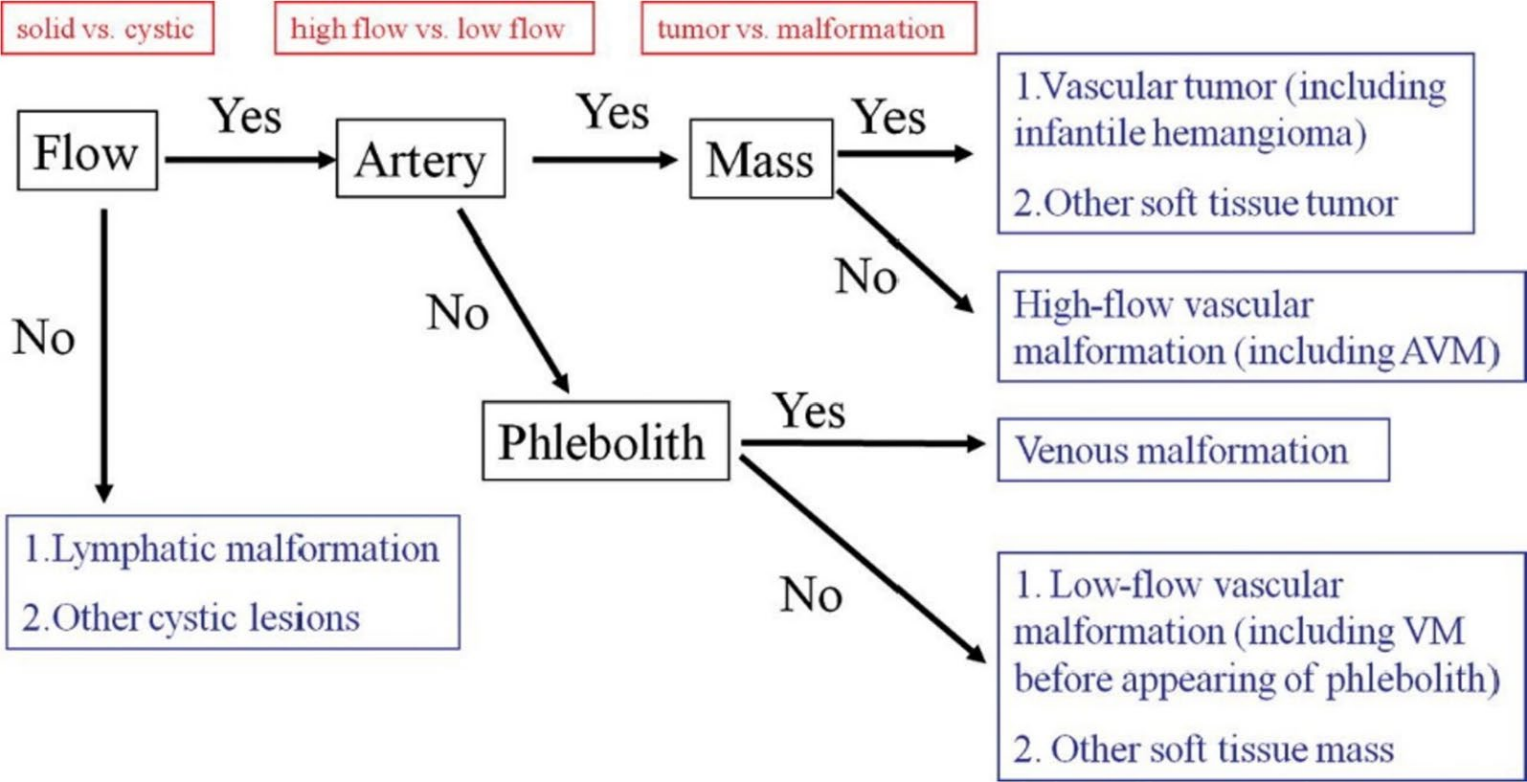
Flowchart shows the classification of vascular malformations. First, the presence or absence of blood flow within the lesion determines whether it is solid or cystic. Next, the presence or absence of arterial components within the lesion determine whether it is high-or low-flow. Finally, the presence of a mass distinguishes tumors from malformations. However, some lesions (e.g., complex malformations) are difficult to classify [5]

Ultrasonography (US) serves a great purpose due to its superficial spatial resolution, ability to allow assessment of fluid and vascularity, lack of ionizing radiation or need for sedation and dynamic capabilities, it is also readily available and relatively of a low cost [3] (Figs. 1, 2).

In this report, we present our experience over the last 2 years in the diagnosis and management of different cases of vascular anomalies.

## Methods

This report included cases of vascular anomalies who were attending the interventional radiology department as well as the vascular anomaly clinic in Abo El-Rish hospitals during the period 2019 through 2021. This study was approved through expedited review by the scientific/ethical committee of the Diagnostic and Interventional Radiology department as well as the ethical committee at the faculty of medicine, Cairo University under the code (D-26-2019).

This study included 60 patients (42 females and 18 male), Ultrasonography and Doppler examination of different vascular anomalies were done as well as interventional or pharmacological treatment. They included females and males. Their ages ranged between 7 days and 50 years.

Our study was an observational prospective study with that a convenient sample of population was taken.

*Inclusion criteria* were any patient having vascular anomaly and *exclusion criteria* included lesions who were previously treated or operated upon.

### Radiological findings: ultrasonographic and Doppler examination

All patients had standardized ultrasonography of the vascular soft tissue swelling with that excess gel was used. Linear high frequency probes were used to perform ultrasound examinations, then color Doppler examinations were performed, examination of the lesions were done with special techniques as Valsalva maneuver, compression of the lesion and limb dependency as needed.

Ultrasound examinations were performed using GE Logic pro 6 and Canon Avio 500 ultrasonographic devices (7–14 MHz) transducers, if treatment was applied, examinations before and after therapy were done, and comparison was performed to delineate the effect of it (measurements of size of the lesion, number of vessels per diameter area, types of flow; low, high or mixed flow, peak systolic velocities, resistivity indices…etc.). The average duration time of the examination ranged between 15 and 20 min, the average follow-up time was 1 month.

In ultrasound imaging of vascular anomalies, B mode imaging is used to define the lesion profile; e.g.,: lesions with a solid appearance in ultrasound imaging are usually vascular tumors, whereas malformations consist of elements with a sponge-like appearance. Color and power Doppler as well as pulsed wave imaging must be used. Color Doppler imaging provides information on the presence of blood flow. Pulsed Doppler imaging reveals information on the hemodynamic characteristics of the vessels of the anomaly [6].

Differentiating venous and lymphatic malformations could be done by clinical background, MR imaging (illustrated in Table 1) and most importantly U/S and Doppler imaging (detailed in the next paragraphs), an overlap between both malformations may be seen in clinical practice with some cases show combined veo-lymphatic malformation, this will not usually affect the treatment plan as both are treated by injection sclerotherapy.

**Table 1:** Clinical and MR imaging features of different vascular anomalies [7]

Vascular anomalies	Clinical features	MR imaging features*	Treatment
Vascular tumors			
Infantile hemangioma	Proliferating phase: occurs in 1st few weeks of life; rapidly growing lesion; strawberry-like, pulsatile, warm mass	Proliferating phase: well-defined lobulated mass, low SI on T1WI, high SI on T2WI, flow voids on SE images, no perilesion edema, early homogeneous enhancement	None (propranolol)
	Involuting phase: grayish dark red mass; complete regression at age 7–10 y	Involuting phase: fat replacement (high SI on T1WI), decreased enhancement	
Low-flow vascular malformations			
Venous	Occurs in childhood or early adulthood; blue, soft, compressible, non-pulsatile mass; grows proportionally with the child without regression	Septated lobulated mass without mass effect, phleboliths (low SI), fluid–fluid levels, low SI on T1WI, high SI on T2WI, no flow voids on SE images, infiltrates tissue planes, surrounding edema possible, no arterial or early venous enhancement, slow gradual enhancement, diffuse enhancement on delayed images	Percutaneous sclerotherapy
Lymphatic	Occurs in childhood; smooth, noncompressible, rubbery mass; grows proportionally with the child without regression	Septated lobulated mass, fluid–fluid levels, low SI on T1WI, high SI on T2WI, no flow voids on SE images, infiltrates tissue planes; if macrocystic, has rim and septal enhancement; if microcystic, no significant or slight diffuse enhancement	Percutaneous sclerotherapy
Capillary	Occurs at birth; cutaneous red discoloration; grows proportionally with the child without regression	Skin-thickness lesion	None
High-flow vascular malformations			
AVM	Occurs in childhood or early adulthood; red, pulsatile, warm mass with a thrill; grows proportionally with the child without regression	No well-defined mass; enlarged feeding arteries and draining veins; flow voids on SE images; infiltrates tissue planes; early enhancement of enlarged feeding arteries and nidus with shunting to draining veins	Transarterial embolization

*the guide reference in our study in the classification of venous and lymphatic malformations compared to ultrasound and Doppler examination

Sonographically, venous malformations appeared as well-margined masses with a heterogeneous echo-structure, the mass is always well compressible. Sometimes, it is possible to identify anechoic tubular structures that are recognized as vascular channels with the presence of a phlebolith (i.e., an intra-lesional calcification) [8].

In the color Doppler examination, venous malformations are slow flow lesions. Vascular density is very low, a light compression on the lesion may be useful to reduce the caliber of the vessels and try to increase the velocity of the intravascular flow [9].

With US the most frequent macrocystic lymphatic malformations appear as lesions containing numerous cystic formations of variable dimensions with liquid content separated by thin hyperechogenic septa, the lesion is deformable, and compression with the probe alters the shape of the cysts [10].

We had not encounter microcystic type of lymphatic malformation in our study.

High flow arterial malformations present at pulsed wave with spectra of high peak velocities and rather high diastolic flow velocities resulting in a low (< 0.5) resistance index (RI) [11].

No definite cut-off values were determined in the literature for RI and PSV differentiating low and high flow malformations.

Other imaging modalities were done as needed, results of U/S and colored Doppler examination were compared to MR imaging in cases of venous and lymphatic malformations, it was compared to angiography either CT or conventional in case of high flow malformations and compared to the clinical data in cases of infantile hemangiomata.

While comparing the categorical data, Chi square test or Fisher's exact test were performed as appropriate, the diagnostic accuracy (sensitivity and specificity) of U/S and other modalities were calculated as well as positive and negative predictive values, *p* values less than 0.05 were considered statistically significant.

### Treatment options

Oral propranolol was given (Indral) at 2–3 mg/kg/day divided into three doses per day, good history taking, electrocardiography (ECG) and echocardiographic examinations were done prior to its administration to excluded cardiac abnormalities, a mean period of 6 months was applied for effective outcomes.

Intra-lesional injection of Betamethasone sodium phosphate 4 mg/Dipropionate 10 mg (Betafos 2 ml ampoule, Egypt) was done in some cases of infantile hemangiomas, total dose varied from 0.1 to 1 cc according to the lesion size, sessions were repeated every other week for average 2–3 times to reach effective results, U/S guidance was performed.

Intra-lesional injection of Bleomycin (Bleocin 15 mg vial)/Albumin (Buminate 20%) mixture in cases of lymphatic and venous malformations was done, the dose was calculated as 0.5 mg/kg body weight not exceeding 10 units at a time, U/S as well as fluoroscopic guidance were done, injection of contrast material iohexol (Omnipaque 300 mg) was done prior to the sclerosing material injection to differentiate the types of venous and lymphatic malformations.

Endovascular embolization using glue (Histoacryl) through micro-catheters was done in cases of high flow vascular malformations, we didn't need to do venous side compression in our cases.

### Interval follow-up

In our study, follow-up of our patients were done by U/S every 1 month after receiving medications and 1 month after intra-lesional injections, unless post-injection complaints were reported.

## Results

This study included 60 patients. They included 42 females and 18 males with their ages ranged between 7 days and 50 years, all of them had soft tissue swellings, suspected to be vascular (Table 2).

**Table 2:** the maximum, minimum, median, mean & standard deviation (SD) of the age

	Minimum	Maximum	Mean ± SD
Age (in years)	0.02	50	12.2 ± 9.4888

**Fig. 3:**
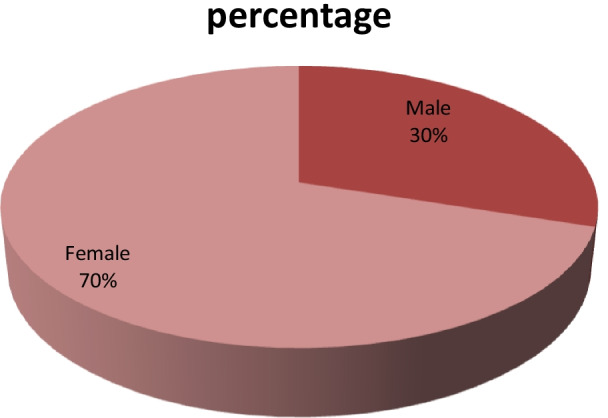
The percentage according to sex

**Fig. 4:**
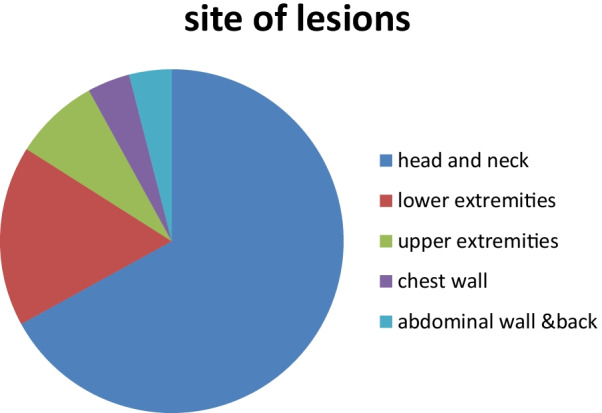
pie chart that delineates sites of different lesion

The frequency and percentage according to sex in the study population are tabulated by a chart, male patients represented 30% while female population represented 70% (Figs. 3, 4; Table 3).

**Table 3:** The distribution of 11 pathological entities, which were diagnosed by all utilized imaging modalities in 60 soft tissue vascular and non-vascular lesions

Pathological entities	Frequency	Percentage (%)
Venous malformations	27	45
Lymphatic malformations	9	15
Infantile hemangioma	8	13.3
Congenital hemangioma	2	3.2
High flow arterial malformation	6	10
Sturge weber	1	1.7
Klippel trenaunay weber syndrome	1	1.7
Skin nevus	3	5
Congenital varicose veins	1	1.7
Angiolipoma	1	1.7
Neurofibroma	1	1.7
Total	60	100

### Venous malformations

**Fig. 5:**
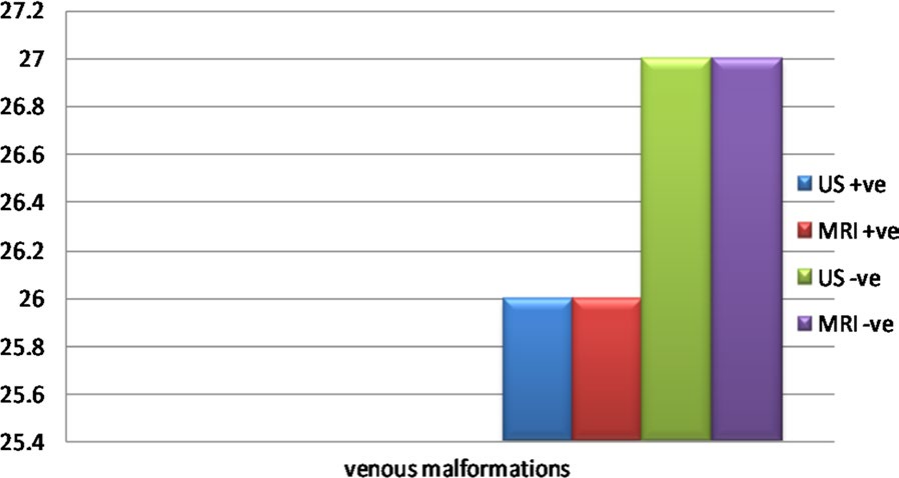
Comparison between US & MRI in the detection of the venous malformations

53 patients did MR and US examinations, ultrasound detected venous malformations in 26 patients with vascular swellings same as MRI imaging with the U/S had superiority in determining the type of flow in venous malformations (while MR could not differentiate), it also helped in the detection of cystic spaces, their compressibility, MR imaging had advantage in the detection of larger and deeper lesions extent (Table 4; Fig. 5).

$$\begin{aligned} & {\text{Sensitivity}} = 100\% ,\;\;\;{\text{Specificity}} = 100\% , \\ & {\text{Positive}}\;{\text{predictive}}\;{\text{value}} = 100\% , \\ & {\text{Negative}}\;{\text{predictive}}\;{\text{value}} = 100\% ,\;\;\;{\text{Accuracy}} = 100\% \\ \end{aligned}$$

**Table 4:** Comparison between US & MRI in the detection of site and exact lesion extensions and characterization of the venous malformations

	Positive cases		Negative cases	
	True	False	True	False
Venous malformations				
U/S	27	0	26	0
MRI	27	0	26	0
Percentage of detection of venous malformation in U/S and MRI	51%	0%	49%	0%

### Lymphatic malformations

Ultrasound detected lymphatic malformations in 9 patients with vascular swellings same as MRI imaging, U/S failed to detect the exact extensions of two patient's that having veno-lymphatic malformations of the orbit; especially in the retro-orbital regions (Table 5).

**Table 5:** Comparison between US & MRI in the detection and characterization of the lymphatic malformations (dilated cystic spaces)

	Positive cases		Negative cases	
	True	False	True	False
Lymphatic malformations				
U/S	7	0	44	2
MRI	9	0	46	0
Percentage of U/S detection of lymphatic malfomation	13.2%	0%	87%	3.77%

Statistical analysis of these results showed significant agreement (*p* value < 0.002) and no significant statistical difference between the two modalities in the detection of lymphatic malformations, U/S have superiority detection of cystic spaces, compressibility, dynamic examination and in the classification of lymphatic malformations, MR imaging had advantage in the detection of deeper and retro-orbital lesions. Sensitivity = 77.8%, specificity = 100%, positive predictive value = 77.8%, negative predictive value = 100%, accuracy = 96.2%

### Arterial malformations

Ultrasound and color Doppler examination detected high flow malformations in 8 patients same as in different angiographic examinations (Table 6).

**Table 6:** Comparison between US & angiography (either endovascular or CT angiography) in the detection and characterization of the high flow malformations

	Positive cases		Negative cases	
	True	False	True	False
Arterial malformations				
U/S	7	0	1	0
Conventional angiography	7	0	1	0
Percentage of detection of arterial malformations in U/S and conventional angiography	87.5%	0%	12.5%	0%

One negative patient was diagnosed as congenital varicose vein and had no arterial flow in Doppler examination nor feeding vessel in conventional angiographic examination, statistical analysis of these results showed significant agreement (*p* value < 0.001).

#### Follow-up by U/S and color Doppler

**Fig. 6:**
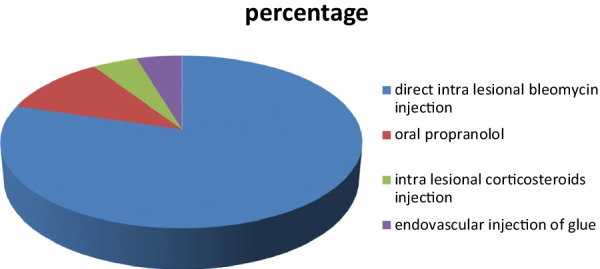
Pie chart showing percentage of treatment plans in treated patients

Follow-up of 43 patients was done after treatment; 34 received bleomycin by intra-lesional subcutaneous injection (U/S guided), and 2 received endovascular sclerosing agent (Glue) (angiographic guided), 5 received propranolol, and 2 received subcutaneous corticosteroids injections (Fig. 6).

## Discussion

Vascular anomalies are classified into vascular tumors and vascular malformations. Vascular tumors demonstrate endothelial cell hyperplasia (commonest is infantile hemangioma) and tend to involute. In contrast, malformations have flattened endothelial cells and do not involutes spontaneously [12].

The ultrasound examination, however, has intrinsic limits. It cannot clearly define the limits in the case of very extensive and deep lesions, and it presents difficulties in exploring some areas such as those near bony and air-filled structures [13].

Cerbu et al. [14] concluded that most used imaging technique for diagnosing vascular anomalies in children is the U/S.

In our study; we aimed to determine the effectiveness and accuracy of U/S and color Doppler examination in evaluation of vascular anomalies, we enrolled 60 patient who met the inclusion criteria, there mean age was 12.2 ± 9.5 years.

### Gender prevalence

Our study included 42 female and 18 male with the ratio 3:1, this agreed with the revised ISSVA 2014 classifications of vascular anomalies which showed same ratio of vascular anomalies seen in male and female patients (mainly in infantile hemangiomata).

### Pathological entities

Our study was comprehensive and enrolled most of common vascular anomalies, in contrast to other studies as in Flors et al. [7], who were more concerned by venous malformations and Ballah et al. [15] who did their study about lymphatic malformations.

### Lesion location

In our study, we found that 67% of the lesions were in the head and neck, 25% of the lesions were in extremities, and 8% were in the trunk.

Flors et al. [7] illustrated that venous malformations occur in the head and neck with 40% percentage, the trunk (20%), extremities encompass approximately 40%.

### Lesions detection and classifications

#### I-Venous malformations

**Fig. 7:**
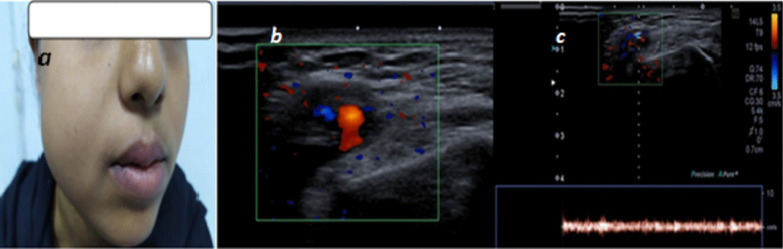
**a** 20-year-old female patient having right facial swelling appeared shortly after birth, no signs of skin inflammation. **b**, **c** US examination of the right aspect of the face showed vascular soft tissue swelling having mixed venous and low flow arterial wave patterns with cystic spaces

**Fig. 8:**
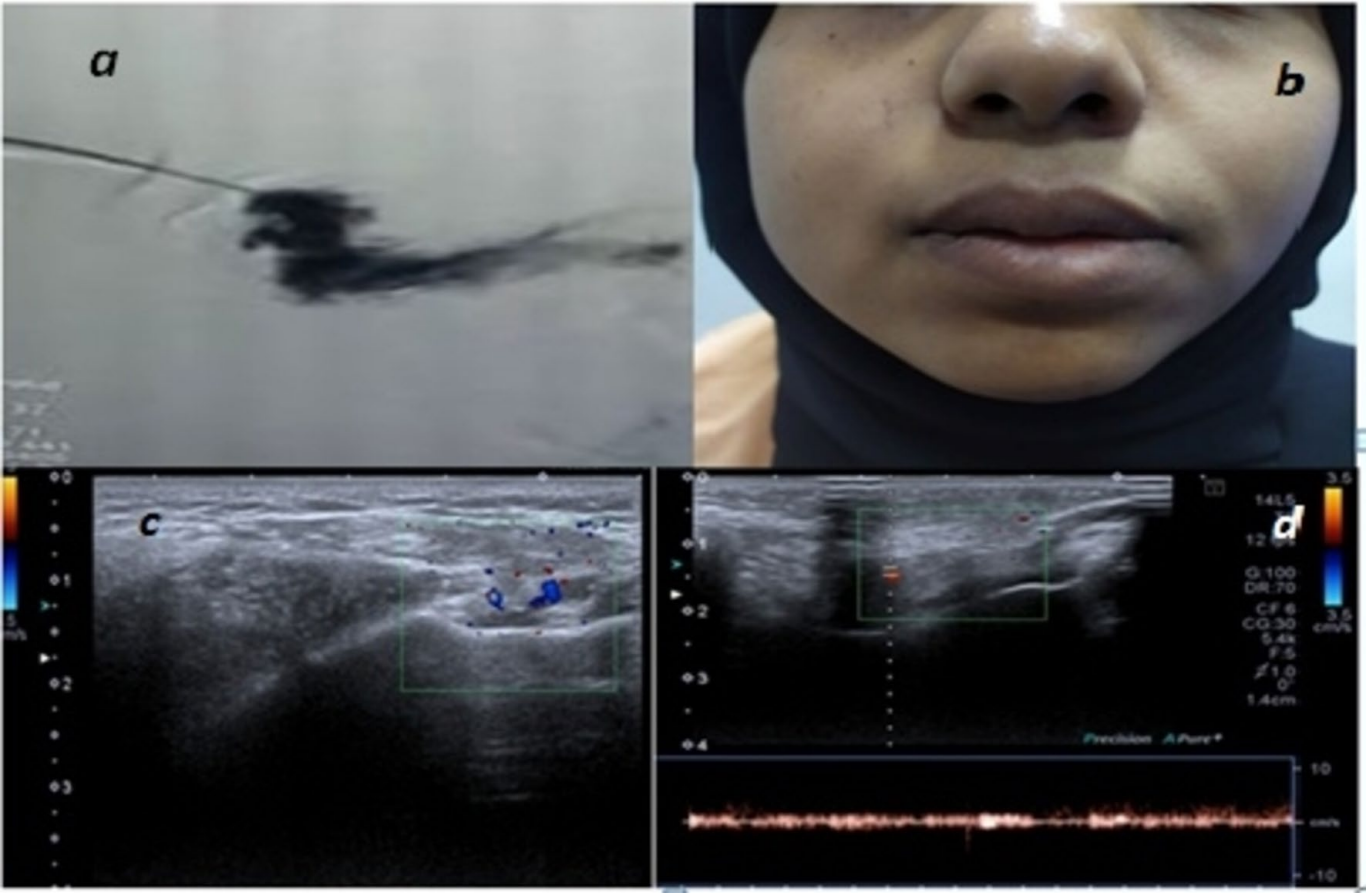
**a** Angiographic appearance of right facial venous hemangioma type I, isolated malformation without venous drainage, **b** 3 month follow-up after injection sclerotherapy, regression of lesion size by 50%, **c**, **d** follow-up U/S images showing: few phleboli of the sclerosed vessels, sclerosed cystic spaces, decreased number of vessels per diameter area (from 5 to 2 vessels/5cm^3^

In our study, we compared the definition of venous malformations between two modalities U/S, color Doppler and MR examinations, we found that U/S can define 100% of lesions dimensions as well as lesions characterization, U/S had the superiority in determining the type of flow either low or high, it had superiority in the detection of cystic spaces and assessing the compressibility of lesions, MR imaging had advantage in the detection of larger and deeper lesions extent, Samadi and Salazar [1]; stated that US evaluation was the first modality for diagnosis of VM, MRI can provide significant information for treatment planning and improvement of symptoms (Figs. 7, 8).

#### II-Lymphatic malformations

In our study, we compared the extent and characterization of lymphatic malformations between U/S and MR examination, we found that U/S have superiority detection of cystic spaces and compressibility of the lesions, it was superior in the classification of lymphatic malformations, MR imaging had advantage in detection of deeper lesions and retro-orbital lesions.

**Fig. 9:**
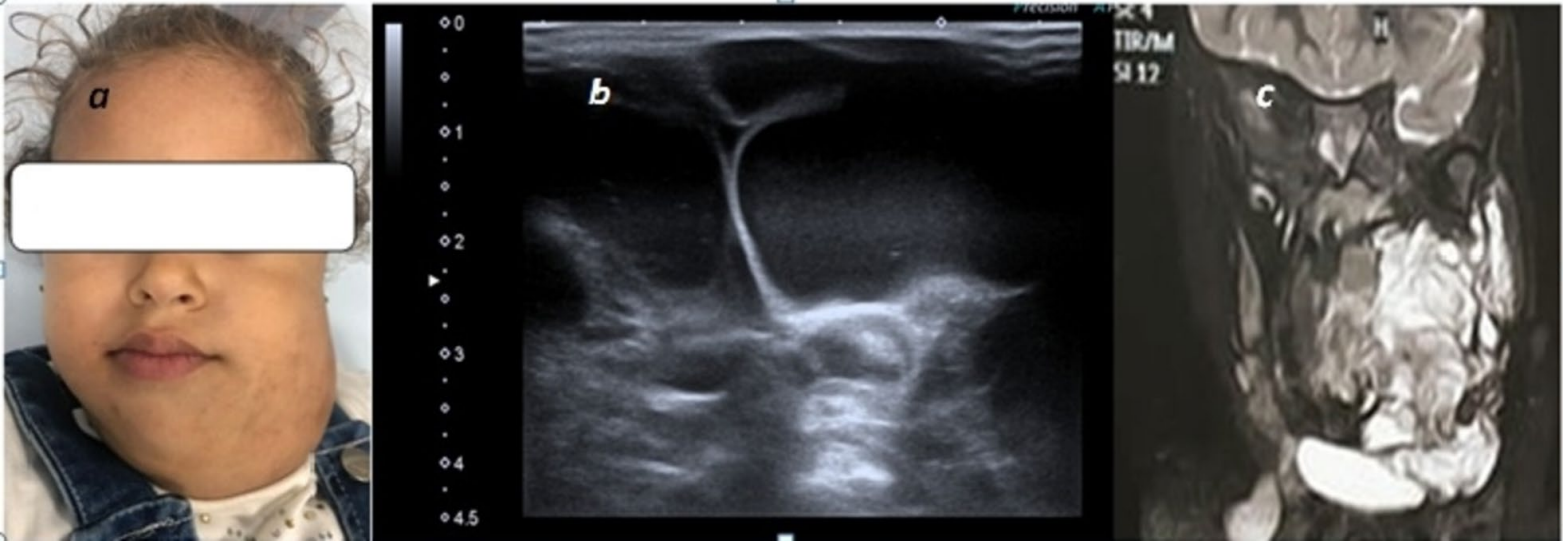
**a** 1.5-year-old female patient came with cystic swelling of the left submandibular region, **b** gray scale images showing multi-locular cystic swelling of the left neck with multiple septations, clear internal fluid, no definite color flow, **c** coronal T2 images showing large cystic swelling extending the left lower face and crossing midline, diagnosis: macrocystic type of lymphatic malformation

**Fig. 10:**
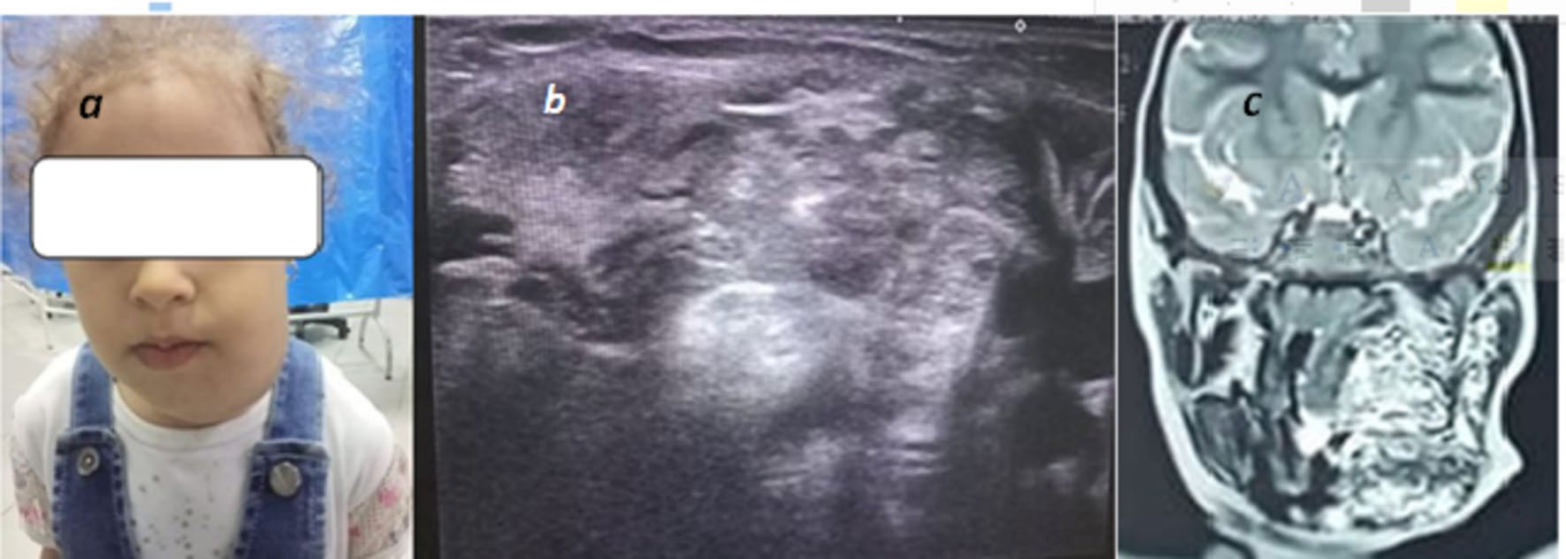
**a** Same patient after staged direct injection sclerotherapy under general anesthesia using bleomycin/albumin mixture injections, (injection was guided by U/S and fluoroscopy), picture of the patient after two and half years showing marked reduction of size, **b**, **c** follow-up U/S and coronal T2 image of the same patient showing nearly completely sclerosed all cystic spaces

Lymphatic malformations were represented in our study and were efficiently seen and diagnosed by U/S imaging (Figs. 9, 10).

#### III. High flow arterial malformations

In high flow arterial malformations, U/S and color Doppler were compared to angiography either CT or conventional, and they agreed in diagnosis of high flow malformations and showing their extensions; yet, angiography showed detailed assessment of the supplying arteries, and interventional angiography was used in therapy, No significant statistical difference between the two modalities in the detection of high flow malformations.

**Fig. 11:**
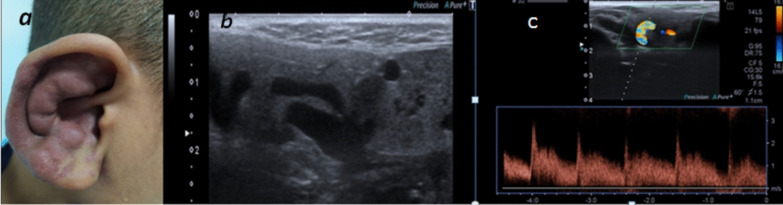
**a** 7-year-old male patient: Image of right auricle showing swelling and discoloration of the right auricle, **b** U/S image showing dilated serpigenous structures, **c** color Doppler of the lesion showing high flow vascular lesion with aliasing artifacts with PSV reaching 2.8

**Fig. 12:**
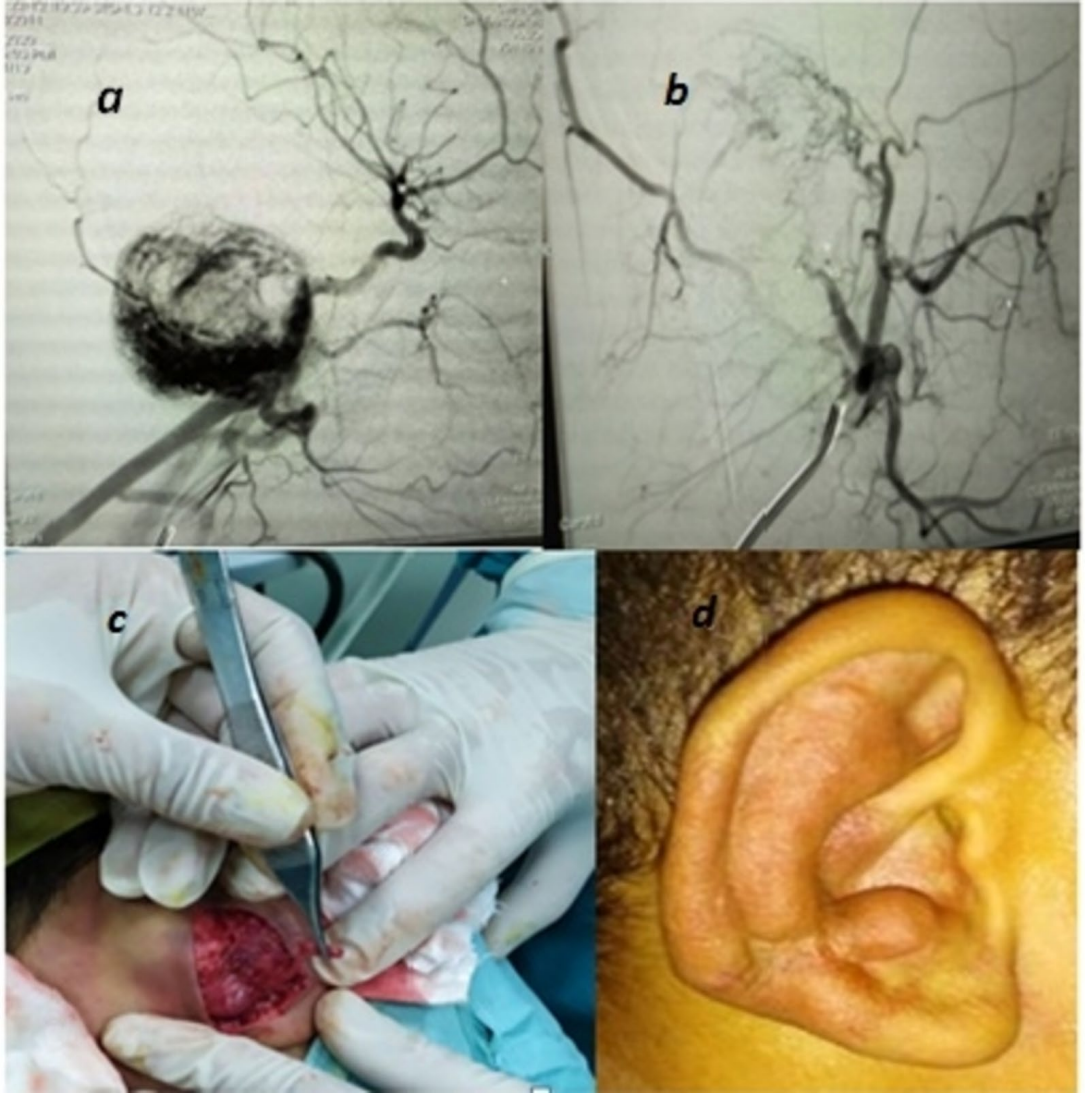
**a**, **b** Same patient: angiographic image of the right external carotid artery, **c** surgical resection by plastic surgery unit following angiographic embolization and **d** follow-up image after 10 months, decrease in lesion size by 50%, disappearance of lesion pulsations and tinnitus, complete surgical resection was not done for fear of skin necrosis

In this study, high flow arterial malformation showed by U/S moderate-sized vessels detected in soft tissues was detected, by color Doppler high flow was seen inside these vessels reaching 2.8 m/s with low resistivity indices (Figs. 11, 12).

#### V. Infantile hemangioma

Correlation between clinical data of infantile hemangioma and US accuracy, US and Doppler examination were able to detect nine cases of infantile hemangioma as well as the two cases of congenital hemangioma.

**Fig. 13:**
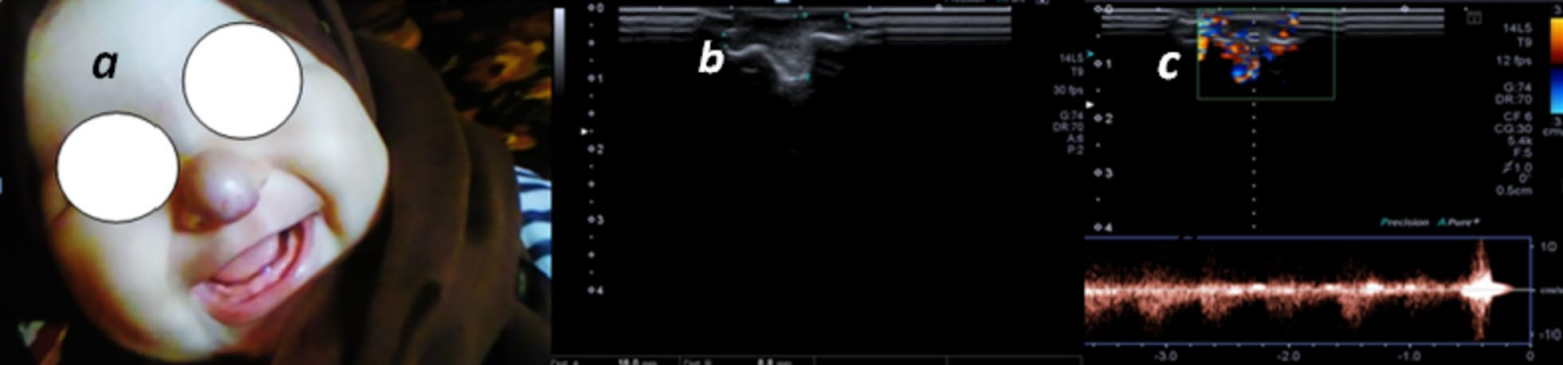
**a** Picture of the patient at age of 4 months showing reddish nasal swelling. **b**, **c** a 1.8 × 0.8 cm nasal swelling was seen showing internal venous and low flow arterial vascularity

**Fig. 14:**
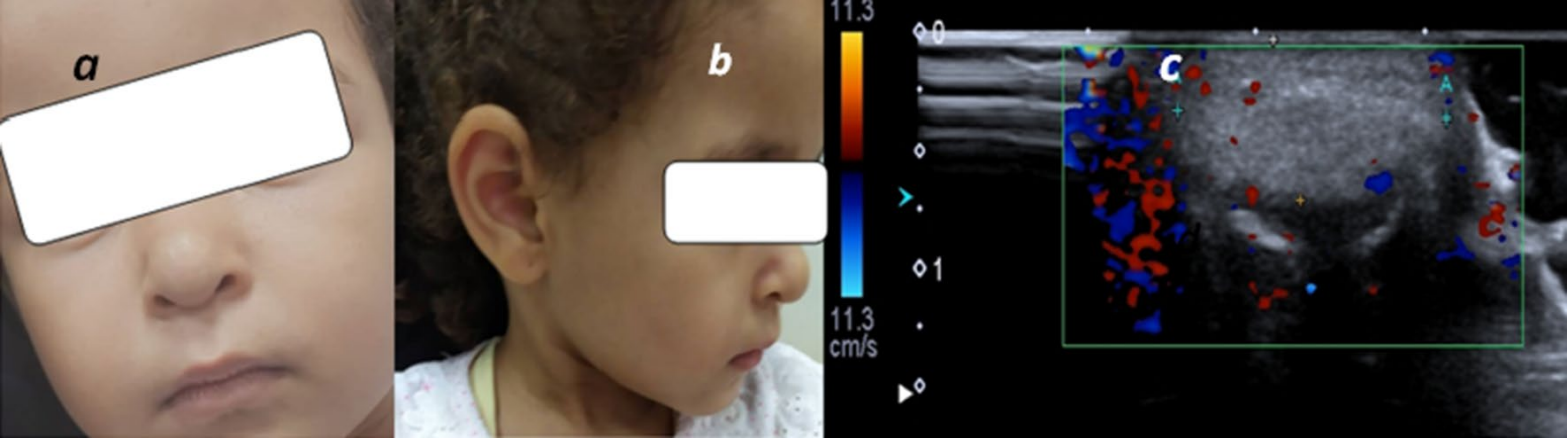
**a**, **b** Follow-up images at the age of three years show marked regression of size and resolution of skin redness, **c** follow-up U/S showed decreased size (1 × 0.5 cm) of lesion as well as its vascularity

Infantile hemangiomata were presented in this study. It showed echogenic soft tissue and increased its internal vascularity in the patient's first visit (proliferative phase), follow-up in this case (after 2 years and 8 months) after oral propranolol treatment showed that the lesion became more echogenic and had subtle residual vascularity, prolonged periods of follow-up were due to COVID 19 lockdown, closing the outpatient clinics and reserving hospitals for only emergency cases (Figs. 13, 14).

In our study, U/S and color Doppler were an efficient tool to exclude swellings that were not vascular in origin; eg: fibrous tumors, dermal lesions, cysts…etc.

### Treatment and follow-up

In this study, follow-up of 43 patients was done after treatment; 34 received bleomycin by intra-lesional direct injection (U/S guided), and 2 received endovascular sclerosing agent (Glue) (angiographic guided), 5 received propranolol, and 2 received corticosteroids injections intra-lesional.

### Interval follow-up

In our study, follow-up of our patients was done by U/S every 1-month post-medications and 1 month post-intra-lesional injections, unless post-injection complaint was reported; as sudden onset of pain, acute redness, hotness progressive enlargement.

Hassan et al. [16] had done follow-up U/S and color Doppler examinations every 3 to 4 weeks; However, Mathur et al. [17] followed their patient's every 2 weeks. From our experience, these variations can vary widely according to patient's education, distance of travelling and interventional radiologist preferences and experiences.

### Complications

U/S and color Doppler examination were efficient in detecting early post-intervention complications. It detected acute superficial thrombo-phlebitis post-sclerotherapy of left pre-auricular venous malformation which was treated conservatively. Skin ulceration/discoloration in case 1 was observed after post-embolization surgical resection; it was treated by regular disinfection and sterile dressing. We observed one case of subacute intra-lesional hemorrhage in case 5 which was aspirated under general anesthesia.

Follow-up by MR was done 1 months in some cases and at the end of treatment sessions in other cases (to limit exposure to general anesthesia, prolonged examinations and to decrease cost and repeated hospital visits for our patients).

### Clinical versus radiological outcome

In our studies, the clinical outcome (pre-and post-procedure photographs and the follow-up notes) in some cases exceeded the radiological outcome (U/S and MR findings), patients had satisfactory results (regarding the cosmetic disfigurement and/or pain) although sizable residual lesions were observed in follow-up U/S and MR images, we believe that this is related to the size of the lesion sclerosed from its superficial prominent component; as the deeper component may be non-visualized and asymptomatic.

## Conclusion

This study showed that U/S and color Doppler examination were effective and accurate methods in diagnosis, classification of superficial soft tissue vascular anomalies, detection of early complications after treatment, follow-up after different treatments applied, it was also beneficial in the exclusion of non-vascular lesions.

Clinical outcomes may proceed radiological outcomes in many cases after treatment of vascular anomalies.

This report may be criticized as the sample size was not large enough for powerful conclusions; other limitations were few patients who were lost to follow-up due to COVID 19 lock down.

Larger scale studies are recommended.

## Data Availability

The datasets used and analyzed during the current study are available from the corresponding author on reasonable request.

## Abbreviations

CT: Computed tomography
EO: Ethanolamine oleate
IH: Infantile haemangiomas
ISSVA: International Society for the Study of Vascular Anomalies
MHz: Mega hertz
MRA: Magnetic resonance angiography
MRI: Magnetic resonance imaging
NO, N: Number
PSV: Peak systolic velocity
SI: Signal intensity
STS: Sodium tetradecylsalfate
US: Ultrasound
VM: Venous malformation
WI: Weighted image
